# A Time Course Analysis of the Conceptual and Affective Meanings of Words

**DOI:** 10.3390/bs15010069

**Published:** 2025-01-15

**Authors:** Dandan Jia, Ling Pan, Mei Chen, Zhijin Zhou

**Affiliations:** 1 School of Management, Henan University of Chinese Medicine, Zhengzhou 450046, China; panling_htc@hactcm.edu.cn (L.P.); chenmei@hactcm.edu.cn (M.C.); 2 School of Psychology, Central China Normal University, Wuhan 430079, China; zhouzj@ccnu.edu.cn

**Keywords:** affective meaning, conceptual meaning, emotion-laden words, priming effect, SOA

## Abstract

Words are the basic units of language and vital for comprehending the language system. Lexical processing research has always focused on either conceptual or affective word meaning. Previous studies have indirectly compared the conceptual and affective meanings of words. This study used emotion-laden words, a special type of dual-meaning word, to directly compare the time course of processing conceptual and affective word meanings. Free association was applied in Experiment 1 to investigate the time course of conceptual and affective meanings in dual-meaning words. The results showed that conceptual-meaning processing was superior to affective-meaning processing. In Experiment 2, the semantic/affective priming paradigm was used to directly compare the time courses of processing conceptual and affective word meanings by manipulating stimulus onset asynchrony (SOA) in different ways. The results showed that semantic and affective priming effects could be obtained under short SOA conditions, with no differences between them. Consistent with Experiment 1, only the semantic priming effect was observed in the long SOA condition. These findings suggest that the conceptual and affective meanings of words have different time courses. The conceptual meaning of words includes automatic and controlled processing, whereas the affective meaning mainly involves automatic processing.

## 1. Introduction

Words are the basic units of language processing and crucial for understanding the language system. A word can convey two types of information. The first is conceptual meaning, which expresses descriptive objective things or actions, such as “fruit”, while the other is affective meaning, which conveys certain evaluative information, such as “happy”. Furthermore, another type of vocabulary exists that can convey both conceptual and affective information (Hu & Liu, 2019; Kövecses, 2003; Liu & Hu, 2013; Pavlenko, 2008). For example, “oppressor” can be considered the descriptive meaning of “emperor”, as well as used to convey the affective meaning of “cruelty”. These are called emotion-laden words (Altarriba & Basnight-Brown, 2011; Kazanas & Altarriba, 2015; Pavlenko, 2008; Zhang et al., 2017). Previous studies have confirmed the existence of conceptual and affective word meanings (Hermans et al., 2001; Hu & Liu, 2019; Jia et al., 2023; Liu & Hu, 2013). However, few studies have directly compared the time courses of conceptual- and affective-meaning processing during word processing.

Some previous studies have explored the time courses of semantic and affective-meaning processing of words through the priming paradigm. Research adopting the semantic priming paradigm has shown that the semantic priming effect appeared only in conditions with short stimulus onset asynchrony (SOA; 30~50 ms) (Hermans et al., 2001; Liu et al., 2010). In contrast, some studies have found that semantic priming effects are observed only for long SOA (Sperber et al., 1979). Other studies have found that semantic priming effects appeared regardless of the length of the SOA (Hu & Liu, 2019; Liu & Hu, 2013). These results indicate that the processing of lexical semantic meanings may be automatic, controlled, or both (Mummery et al., 1999; Neely, 2012; Rossell et al., 2003). To date, the results regarding semantic word processing have been inconsistent. One study found that the affective priming effect usually appeared under short SOA conditions (less than 300 ms), indicating that the processing of affective word meaning may be automatic (Hermans et al., 2001; Liu et al., 2010). Some comparison studies have demonstrated significant semantic and affective priming effects under both long and short SOA conditions (Ferré & Sánchez-Casas, 2014). In research on priming the conceptual and affective meanings of words, the relationship between them has been mainly explored by indirectly comparing the priming effects of both; however, these findings are unreliable. In the semantic priming study, the experimental materials were mainly neutral nouns and semantically related tasks, while in the affective priming study, the experimental materials were mainly emotional words, and the experimental tasks were mainly emotion-related tasks. Thus, the results are difficult to objectively compare, and the research findings on the processing of conceptual and affective word meanings remain ambiguous. To address this issue, this study aimed to directly compare the relationship between the conceptual and affective meanings of words. In one experiment, the same materials and experimental paradigm were used to directly compare both processing methods.

Emotion-laden words are typical dual-meaning words with both conceptual and affective meanings (Jia et al., 2023; Pavlenko, 2008; Zhang et al., 2017). For example, the word “oppressor” has both a conceptual meaning of “emperor” and an emotional meaning of “cruelty”. Emotion-laden words provide excellent experimental materials for direct comparisons between conceptual and affective meanings. Thus, the present study aimed to directly compare affective and conceptual word meanings through two experiments to explore the time course of their processing. Experiment 1 adopted the free association method to explore whether the time course for active processing differs for the conceptual meaning and affective meaning of emotion-laden words, which also provided experimental materials for Experiment 2. In Experiment 2, the semantic/affective priming paradigm was used to directly compare the time courses of processing words’ conceptual and affective meanings by manipulating the SOA (50 ms, 400 ms). Four conditions were constructed for the experiment simultaneously by taking the emotion-laden words (e.g., “oppressor”) as the target stimuli: semantic priming condition, semantic control condition, emotional priming condition, and emotion control condition. When the prime word was related to the conceptual meaning of the target word, it constituted the semantic priming condition (e.g., “emperor”—“oppressor”). When the prime word was unrelated to the conceptual meaning of the target word but belonged to the conceptual-meaning word, it constituted the control condition of semantic priming (e.g., “animal”—“oppressor”). When the prime word was related to the affective meaning of the target word, it constituted the affective priming condition (e.g., “cruelty”—“oppressor”). Meanwhile, when the prime word had no relation with the emotional meaning of the target word but belonged to the common emotional word, it constituted the affective priming control condition (e.g., “selfish”—“oppressor”).

In general, we set up corresponding control conditions for both the conceptual and emotional processing of word meanings. In the semantic priming condition (“emperor”—“oppressor”), the conceptual meaning of “oppressor” was activated by the word “emperor”. Corresponding to the semantic priming control condition (“animal”—“oppressor”), the prime word had a conceptual meaning and belonged to the same lexical category as the prime word in the semantic priming condition, with the only difference being that it was not related to the target word. Similarly, the affective priming condition (“cruelty”—“oppressor”) was designed to activate the emotional meaning of “oppressor” through the word “cruelty”. Corresponding to the affective priming control condition (“selfie”—“oppressor”), the prime word was emotional, with the only difference being that it was not related to the target word. Through this experimental setting, we could not only test the priming effects of lexical conceptual and affective meanings but also compare them directly. Simultaneously, irrelevant words and pseudowords were used to fill in the materials. This experiment used a lexical decision task that required participants to evaluate whether the target was a Chinese word.

This task was biased towards neither the semantic nor affective meaning of words, which was suitable for this study. Lexical decision tasks have been widely used in previous studies (Copland et al., 2003; Rossell et al., 2003).

## 2. Experiment 1

The free association method was used to generate a corpus of the conceptual and affective meanings of dual-meaning words. Meanwhile, the free association process was analyzed, and the order of the conceptual- and affective-meaning associations of dual-meaning words was preliminarily explored.

### 2.1. Method

#### 2.1.1. Participants

We recruited 64 college students (44 female and 20 male) for this experiment. All were 18–25 years old, with an average age of 20.17 (*SD* = 1.32), and were native Chinese speakers with normal or corrected-to-normal vision. As our study was primarily interested in examining the effects of emotional stimulation in the general population, all participants completed the Beck Depression Inventory-II (Grothe et al., 2005) and State-Trait Anxiety Inventory (Spielberger et al., 1971), and only those with scores below 13 and 43, respectively, were included.

This study was approved by the Ethics Committee of the School of Psychology at Central China Normal University and conducted in accordance with the Declaration of Helsinki. All participants voluntarily took part in the experiment and provided signed informed consent. All participants received a monetary reward at the end of the experiment.

#### 2.1.2. Materials

A total of 100 two-character Chinese emotional words with dual meanings were selected from the Chinese Affective Information Assessment words system (Wang et al., 2008). In this study, only dual-meaning words associated with negative emotions were selected because negative information has a high level of arousal and important ecological significance for human survival and adaptation (Peng & Zhou, 2005). To balance the familiarity of all the words, 20 college students who did not participate in the formal experiment were recruited to evaluate the familiarity on a 9-point Likert scale. Finally, we selected 80 dual-meaning negative emotion words (familiarity: *M* = 8.02, *SD* = 0.53) for the formal experiment.

#### 2.1.3. Experimental Procedure

First, the participants were presented with the instructions and asked to make free associations according to the dual-meaning words shown and record them in the order in which each association came to mind. Participants were told to provide at least four words through free association for each word presented.

In the experiment, the experimental material was divided into two blocks. Participants randomly selected one of the blocks. The material was presented on a computer screen, and participants were asked to fill out an online form to record the results. First, the selected words were presented on the computer screen, and then participants made free associations according to the prompted words and recorded them in the table in the order of free association.

### 2.2. Results

Two participants who did not generate dual-word words as required were eliminated. Thus, the results from 62 participants were included in the statistical analysis.

Scoring procedures and criteria in this study were established for all evaluations of the results. The associated words generated by each dual-meaning word were classified as either conceptual- or affective-related words. The conceptually related words were mainly those associated with the conceptual meanings of dual-meaning words. For example, “oppressor” is conceptually related to words such as “emperor” and “monarch”. The affective-related words were mainly words related to the affective meaning of “oppressor”, such as “cruelty” and “abuse”. Five experts were recruited to statistically evaluate the results. All five experts were PhD candidates in psychology who specialized in psycholinguistics. All experts were unaware of the study’s purpose and did not participate in the study.

All words generated through free association were classified to determine whether the first, second, third, and fourth words participants identified as being associated with each dual-meaning word were conceptual or affective meaning-related words. The word categories generated in a different order were statistically analyzed.

A consistency analysis was conducted on the percentage of conceptual and affective meanings in different sequences of each dual-meaning word as assessed by the five experts, and the results showed that Cronbach’s alpha was 0.931. We selected the average percentage of five raters as the final statistical index. The association words of all dual-meaning words were classified according to order and type, and the percentage differences were statistically analyzed. The results are presented in Table 1.

The results showed significant differences between conceptual and affective meanings in different orders of association (*p* < 0.001). This shows that there was a difference in the time course between the two groups.

**Figure 1:**
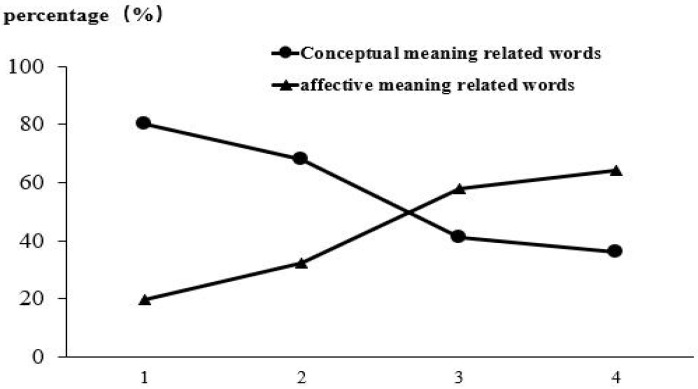
Trend chart of association word types in different orders.

A repeated-measures ANOVA was conducted for the percentage of words that were related to conceptual meanings in order. The results showed that the main effect of order was significant, *F*(2, 60) = 127.294, *p* < 0.001, *η_p_*^2^ = 0.914. Post hoc comparisons showed no significant differences between the third and fourth words (*p* > 0.05). All other conditions were significantly different (*p* < 0.001). A repeated-measures analysis of variance (ANOVA) was performed for the percentage of words related to the affective meanings in different orders of association. The results showed that the main effect of order was significant, *F*(2, 60) = 120.67, *p* < 0.001, *η_p_*^2^ = 0.910. Post hoc comparisons showed no significant differences between the third and fourth words (*p* > 0.05). All other conditions were significantly different (*p* < 0.001). Figure 1 shows the different types of related words in the free association change trends over time.

The results of this experiment showed that, when engaging in free association for dual-meaning words, participants initially associated them with words related to the conceptual meaning, thus processing the conceptual meanings first and then processing the dual-meaning words’ affective meanings second.

## 3. Experiment 2

The results of Experiment 1 show that the processing of dual-meaning words in the free-association stage tends to prioritize conceptual meaning. Based on Experiment 1, this study used experimental methods to further explore the time course of the conceptual- and affective-meaning processing of dual-meaning words.

### 3.1. Method

#### 3.1.1. Participants

We recruited 58 healthy college students (20 male and 38 female) for this experiment. All were 18–24 years old, with an average age of 21.01 (*SD* = 1.55), and native Chinese speakers with normal or corrected-to-normal vision. All participants received a monetary reward at the end of the experiment.

#### 3.1.2. Experimental Design

This experiment used a 2 (priming condition: priming, controlling) × 2 (priming type: conceptual meaning, affective meaning) × 2 (SOA: 50 ms, 400 ms) three-factor design.

#### 3.1.3. Stimuli and Procedure

***Materials.*** The experimental materials included four types of word pairs: conceptual priming word pairs (e.g., emperor–oppressor), conceptual controlling word pairs (e.g., student–oppressor), affective priming word pairs (e.g., cruelty–oppressor), and affective controlling word pairs (e.g., haughty–oppressor). In all the experimental materials, the prime words were neutral and emotional words, and the target words were dual-meaning words or pseudowords. This study primarily selected negative dual-meaning words. Negative emotion words are considered to evoke a high level of arousal and have important ecological significance for human survival and adaptation (Peng & Zhou, 2005). In the experiment, the target words were 60 dual-meaning negative emotional words and 120 pseudowords. The prime words were related to either the conceptual or emotional meaning of the target word. The pairs of conceptual-meaning prime words included highly semantically related prime and target words. Regardless of the glyphs, pronunciation, or emotional meaning, the words were completely unrelated. The correlation degrees of the conceptual meanings were determined by 20 individuals who did not participate in the formal experiment and were evaluated on a 5-point Likert scale (1 = not at all semantically associated; 5 = strongly semantically associated). The average conceptual-meaning correlation degree of the selected word pairs was 4.09, whereas for the conceptual-meaning control word pairs, no relationship was found between each prime and target word, regardless of the semantics, glyph, pronunciation, or emotional meaning. In the pair of affective-meaning prime words, the prime and target words had highly relevant emotional meanings. In addition, no correlation was found regardless of semantic, glyph, or phonetic correlations. The correlation degrees of the affective meanings were determined by 20 individuals who did not participate in the formal experiment and were evaluated on a 5-point Likert scale (1 = not at all affectively associated; 5 = strongly affectively associated). The average affective-meaning correlation degree of the selected word pairs was 4.19, while no relationship was found between the prime and target words in the affective-meaning control word pair. To balance the responses of “yes” or “no” to the target word in the lexical decision task, two real Chinese characters were randomly combined to produce 120 Chinese pseudowords. The Chinese pseudowords (mean strokes: 18.00) and real Chinese words (mean strokes: 19.61) did not differ significantly between strokes, *F*(1, 238) = 5.09, *p* = 0.18.

The words and pseudowords were designed by crossing. Each word appeared once in the priming condition and the corresponding control condition, which ensured the purity of the direct comparison between the priming and corresponding control conditions, thus effectively controlling for the repeated priming effect.

***Procedure.*** The participants completed a lexical decision task to determine if a two-character word was a real Chinese word. Lexical decision tasks have been widely used in previous priming studies (Copland et al., 2003; Mummery et al., 1999). In the present study, this task was not biased towards either the semantic or emotional meaning of the word.

The experiment was conducted in a soundproof room. Participants were seated in a comfortable chair approximately 75 cm from the screen. First, the participants went through a training block to familiarize themselves with the process. All stimuli were presented as white characters (font: Song typeface, size: 58 points) at the center of the computer screen on a black background.

A white fixation point (“+”) was presented at the center of the screen for 150 ms, followed by an empty screen for 400~500 ms. Then, a prime word was presented at random for 50 ms or 400 ms in the short and long SOA conditions, respectively. When the prime word disappeared, the target word was presented immediately, and participants were asked to determine whether the two-character word was a real Chinese word. If the target word was a real two-character word, participants were to press the “F” key; otherwise, they were to press the “J” key. The participants were required to respond within 2000 ms. The two keyboards were balanced among the different participants.

### 3.2. Results

The data for participants’ response times and error rates were analyzed using SPSS 10.0. Trials with error rates higher than 5% and response errors were eliminated, which accounted for 2.43% of the total data. Two participants were excluded from this study, resulting in 56 effective participants. Table 2 shows the responses for the different lexical types under the SOA conditions.

A 2 × 2 × 2 repeated measures ANOVA was performed for condition (priming, control), type (conceptual meaning, affective meaning), and SOA (50 ms, 400 ms). The results showed that the main effects of condition, *F*(1, 53) = 31.24, *p* < 0.001, *η_p_*^2^ = 0.031, type, *F*(1, 53) = 28.31, *p* < 0.001, *η_p_*^2^ = 0.028, and SOA, *F*(1, 53) = 4.11, *p* = 0.043, *η_p_*^2^ = 0.004, were all significant. Moreover, significant interactions were observed between condition and type, *F*(1, 53) = 7.38, *p* = 0.007, *η_p_*^2^ = 0.007, and condition and SOA, *F*(1, 53) = 5.03, *p* = 0.025, *ηp*^2^ = 0.005. No other conditions were significant (*p* > 0.05). Based on this, the response times under the two SOA conditions were analyzed.

**Figure 2:**
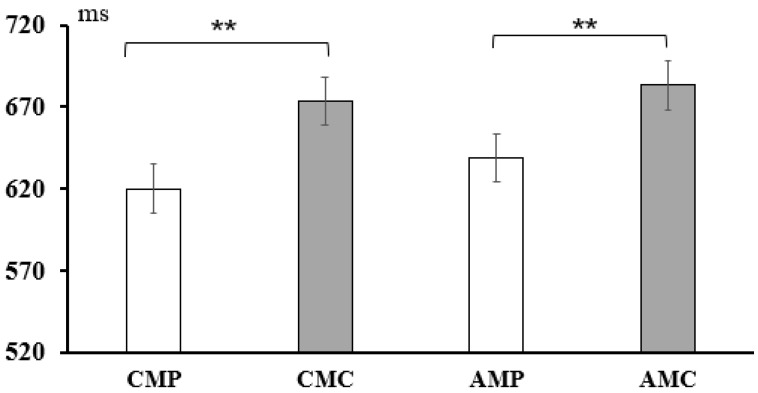
Statistical results of different conditions under short SOA (50 ms) condition. Note: CMP: There was a conceptual-meaning correlation between the prime word and the target word; CMC: Prime words were conceptual words but were not semantically related to target words. AMP: There was an affective-meaning correlation between the prime word and the target word. AMC: Prime words were emotional words, but had no emotional meaning related to target words. Note: Statistically significant differences are marked by *; ** *p* < 0.01.

**Figure 3:**
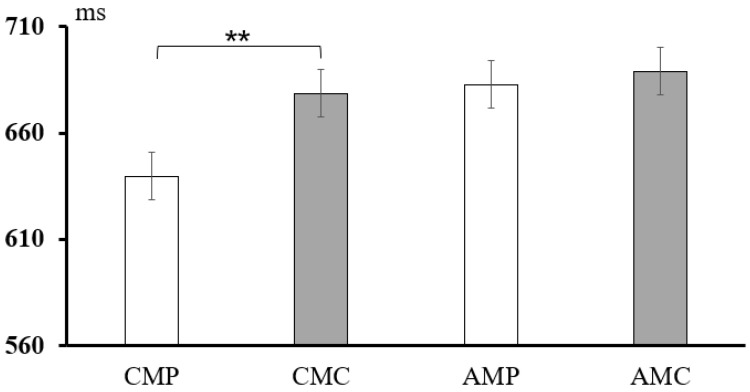
Statistical results of different conditions under long SOA (400 ms) condition. Note: Statistically significant differences are marked by *; ** *p* < 0.01.

In the short SOA (50 ms) condition, a 2 × 2 repeated-measures ANOVA was conducted for condition (priming, control) and type (conceptual meaning, affective meaning). The results showed a significant main effect of condition, *F*(1, 54) = 31.44, *p* < 0.001, *η_p_*^2^ = 0.031. Post hoc comparisons showed that the control condition (*M* = 678.21, *SD* = 111.89) was significantly longer than the priming condition (*M* = 629.41, *SD* =125.09), regardless of the conceptual or affective meaning. No main effects were observed for type, *F*(1, 53) = 2.08, *p* = 0.064, and the interaction between condition and type was not significant, *F*(1, 53) = 0.54, *p* = 0.461. For the long SOA (400 ms) condition, a 2 × 2 repeated-measures ANOVA was conducted for condition (priming, control) and type (conceptual meaning, affective meaning). The results showed a significant main effect of condition, *F*(1, 54) = 7.12, *p* = 0.008, *η_p_*^2^ = 0.007, and types, *F*(1, 54) = 32.12, *p* < 0.001, *η_p_*^2^ = 0.031. Moreover, a significant interaction was observed between condition and type, *F*(1, 54) = 12.43, *p* < 0.001, *η_p_*^2^ = 0.012. Post hoc comparisons revealed that the response times in the priming condition were significantly faster than those in the control condition for processing the conceptual meaning of words (*p <* 0.001). In contrast, no significant difference in response times was found between the priming and control conditions for processing affective word meanings (*p* = 0.52). The results are shown in Figure 2 and Figure 3.

## 4. General Discussion

The present study used a special type of dual-meaning words, emotion-laden words, to directly compare the processing of conceptual and affective meanings of words. Experiment 1 applied a free-association method to analyze the processing of dual-meaning words. The results showed that participants processed the conceptual meaning first and the emotional meaning second. Thus, they processed the semantic meaning first. Based on Experiment 1, we conducted Experiment 2 using a priming paradigm to directly compare the time courses of the conceptual and affective meanings of dual-meaning words. The results showed that the two methods have different processing modes.

Experiment 1 showed that the semantic information of words was more easily activated during conscious active processing. For the free association of dual-meaning words with conceptual and affective meanings, participants first associated semantically related words and then emotion-related words. This supports the theory that lexical semantic information is preferentially processed. Previous studies also used the free association approach to study semantic processing (Spätgens & Schoonen, 2020). In word-free association, semantic information was the first thing to be associated, proving that semantic information had a certain priority in word processing. It was also found in some studies that the free association task of words would be affected by age (Spätgens & Schoonen, 2020), reading ability (Spätgens & Schoonen, 2019), context (Spätgens & Schoonen, 2018), and other factors. Therefore, the participants recruited in this study were all college students, and there was no significant difference in educational background and reading ability, which made the results more universal.

Experiment 2 used an experimental method to explore the time course of conceptual and affective meanings of words by manipulating SOA. The results showed that dual-meaning word processing resulted in significant semantic priming effects in both the short (50 ms) and long (400 ms) SOA conditions, which is consistent with the results of previous semantic priming studies (Hu & Liu, 2019; Mummery et al., 1999; Neely, 2012; Rossell et al., 2003). The results showed that processing the conceptual meaning of words includes both automatic and controlled processing. The semantic priming effect in the short SOA condition (50 ms) indicates an automatic spreading activation mechanism using nodes in the semantic memory network for pre-activation. The semantic priming effect in the long SOA condition (400 ms) reflected a controlling and strategic processing mechanism that was expected through the prelexical phase (Neely, 2012). In conclusion, these results indicate that processing the conceptual meanings of words includes both automatic and controlled processing.

The results of Experiment 2 also show that the affective priming effect appeared only in the short SOA (50 ms) condition. These results are consistent with those of previous studies on affective priming (Hermans et al., 2001; Hu & Liu, 2019; Jiang et al., 2016). The affective priming effect possibly only appeared in the short SOA condition because participants could process emotional information “automatically” (Hermans et al., 2001). This automatic processing occurs in the early stages of information processing, and multiple emotional stimuli can be processed in parallel. This type of processing is fast, aimless, unconscious, and efficient (Zhang et al., 2017; Peng & Zhou, 2005). Rapidly and accurately processing the emotional information in language has great social significance. Under the long SOA condition, emotional activation dissipated, and emotional effects were suppressed (Hermans et al., 2001). Thus, the target stimulus in the longer SOA condition was presented too late to demonstrate the contribution of the priming stimulus to the response. Moreover, we found that the semantic and affective priming effects were both significant in the short SOA condition, with no significant differences between them. The results showed that both conceptual and emotional meanings are important attributes of words and processed rapidly, supporting Bower’s connect-network model. According to this model, emotion and cognitive nodes are connected through a unified connection network, and emotion is not an appendage of cognitive activities but an important part of people’s perception and representation of the world (Bower, 1981).

The present study used dual-meaning words to directly compare the modes used to process the conceptual and affective meanings of words. The results of Experiments 1 and 2 both found that the conceptual-meaning processing of words included both automatic and controlled processing, whereas the affective-meaning processing only included automatic processing. This provides a theoretical basis for the time course of conceptual and affective meanings in vocabulary.

## 5. Conclusions

In this study, emotion-laden words were selected as experimental materials, and the time processes of conceptual- and emotional-meaning processing were directly compared. In Experiment 1, the free association method was used to examine the time courses for conceptual and emotional meanings in emotion-laden words. The results showed that conceptual-meaning processing was preferred over emotional-meaning processing. In Experiment 2, the semantic/affective priming paradigm was used to directly compare the time courses for processing words’ conceptual and affective meanings by manipulating the SOA. The results showed that semantic and affective priming effects could be obtained in the short SOA condition, with no differences between them. In the long SOA condition, only the semantic priming effect of words was observed, which is consistent with the results of Experiment 1. In conclusion, these findings suggest that the conceptual and affective meanings of words have different time courses. The conceptual meaning includes automatic and controlled processing, whereas the affective meaning mainly involves automatic processing. When processing emotion-laden words, emotional information only occurs in the early stages, whereas semantic information continues throughout the process.

## Figures and Tables

**Table 1:** Classification and analysis results of free association of dual-meaning words in different orders.

Free Association Order	Percentage of Association Words of Different Types (%)		*t*	*p*
	Conceptual Meaning-Related Words	Affective Meaning-Related Words		
1	80.27	19.73	18.22	0.000 **
2	67.78	32.22	8.52	0.000 **
3	41.02	57.98	−3.80	0.001 **
4	36.06	63.94	−4.322	0.000 **

Note: Statistically significant differences are marked by *; ** *p* < 0.01.

**Table 2:** Results of response time between four types of target words under different SOAs.

SOA	Conceptual Meaning			Affective Meaning		
	Priming	Controlling	Priming Effect	Priming	Controlling	Priming Effect
50 ms	620.14 (101.18)	673.21 (111.12)	53.07 **	638.68 (174.96)	683.21 (169.76)	44.53 **
400 ms	639.68 (156.83)	678.77 (123.23)	39.09 **	682.89 (137.67)	688.98 (170.45)	6.09

Note: The data in brackets are standard deviations (SD); Statistically significant differences are marked by *; ** *p* < 0.01.

## Data Availability

Data will be made available on request. The data that support the findings of this study are available on request from the corresponding authors.
